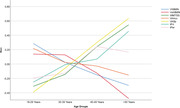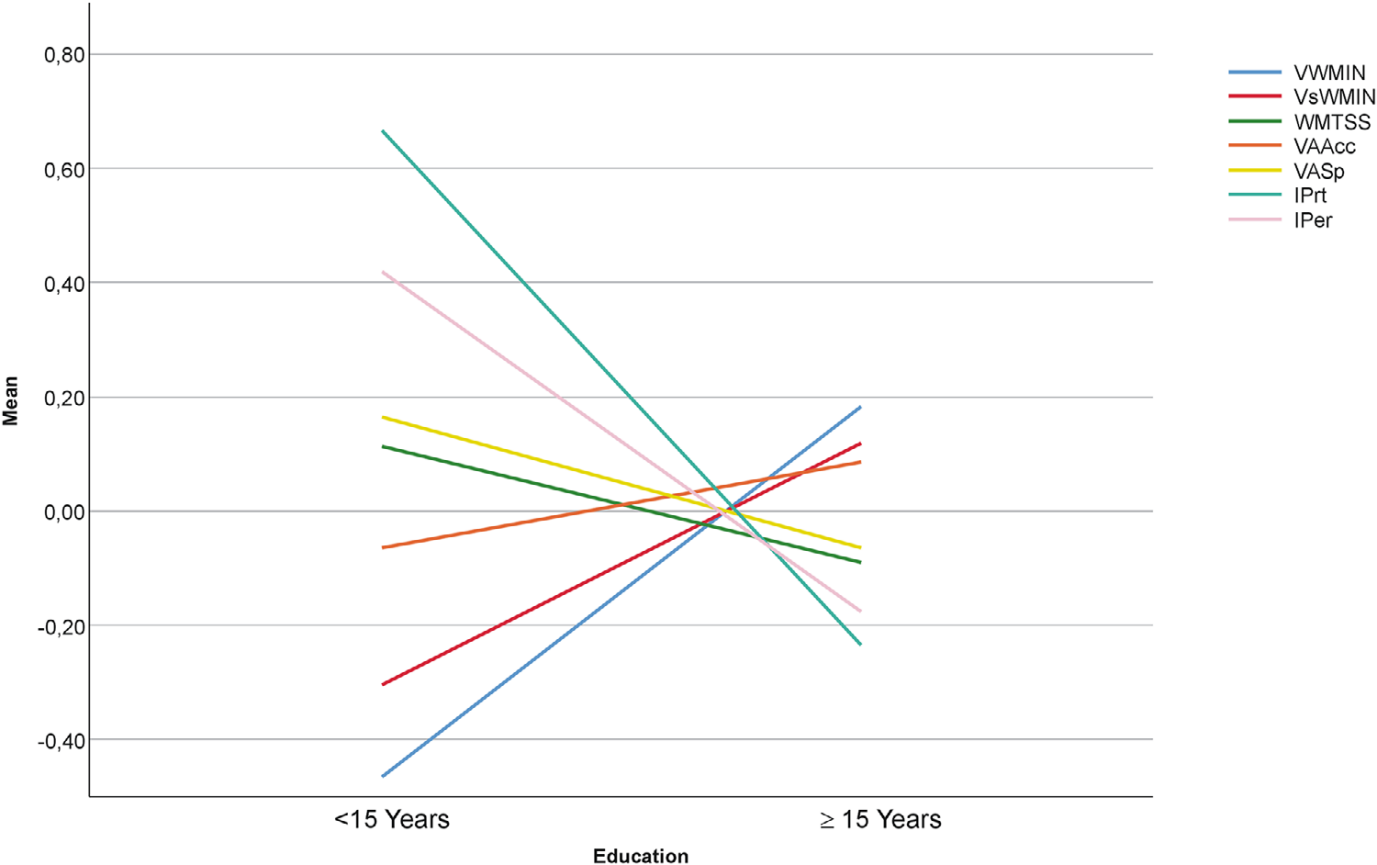# Validation Study of A Novel VR‐Based Cognitive Assessment Tool: Influences of Demographic Characteristics

**DOI:** 10.1002/alz.093073

**Published:** 2025-01-03

**Authors:** Deniz Yerlikaya, Erdil Arsoy, Murat Sükuti, Meliha Beste Baytimur, Eda Özbilgen, Seray Şenyer, Emre Baytimur, Dilek Betül Sarıdede, Simay Bayatlı, Adil Deniz Duru

**Affiliations:** ^1^ Neo Auvra® Digital Health and Bionic Technologies and Services Inc., İstanbul, Üsküdar Turkey; ^2^ Marmara University, Institute of Health Sciences, İstanbul, Kartal Turkey; ^3^ Keio University, Graduate School of Media Design, Tokyo, Tokyo Japan; ^4^ Atlas University, Department of Biomedical Engineering, İstanbul, Kağıthane Turkey; ^5^ Marmara University, Faculty of Sport Sciences, İstanbul, İstanbul Turkey

## Abstract

**Background:**

Emerging technologies paves the way for the development of novel cognitive assessment approaches. Virtual reality (VR) has high ecological validity, increases participant engagement, and allows the assessment of functions that are challenging to evaluate with traditional methods. The present study aimed to validate a novel cognitive assessment tool and investigate the effects of demographical variables.

**Method:**

A total of 575 healthy individuals aged between 18‐66 years were included in the study. A subgroup of the participants (n = 326) attended the verbal and visual versions of the computerized N‐back task. All participants underwent the modified version of NORA VRx™ ‐ Core experience. Seven metrics were calculated from working memory, attention, and information processing tasks during the experience. The relative contributions of the age, education and gender variables on the VR‐based metrics were assessed with multiple linear regression analysis. The concurrent validity was investigated by the correlation between VR‐based scores and N‐back scores using the Pearson Correlation.

**Results:**

Age, gender, and education were significant predictors of the working memory scores, whereas only age and education were the significant predictors for the information processing scores. All the calculated cognitive scores worsened with the increasing age and lower education levels. Male had better scores in working memory and attention measures than female. The correct answer number at the verbal and visual N‐back tasks showed moderate correlations with the VR‐based scores. The working memory scores showed the strongest correlations with the 2‐ and 3‐back conditions (p<0.001 for all).

**Conclusion:**

The present study introduces a novel and valid cognitive assessment tool. The sample was stratified according to age and education and norm values were prepared accordingly. In the future studies, it is important to determine the characteristics of the population aged 50 years and older and to examine the discriminatory power of the novel tool among various patient groups.